# (St)aging in place: Information and communication technologies for a health-centered agile dwelling unit

**DOI:** 10.3389/fpubh.2023.1057689

**Published:** 2023-01-27

**Authors:** Nicole Cevallos, Widya A. Ramadhani, Julia Lindgren, Bradley Bell, Maria Martinez-Cosio, Thomas E. Harvey, Upali Nanda, Gabriela Mustata Wilson

**Affiliations:** ^1^Multi-Interprofessional Center for Health Informatics, The University of Texas at Arlington, Arlington, TX, United States; ^2^School of Architecture, University of Illinois Urbana-Champaign, Champaign, IL, United States; ^3^Center for Advanced Design Research and Evaluation, Dallas, TX, United States; ^4^College of Architecture, Planning, and Public Affairs, The University of Texas at Arlington, Arlington, TX, United States; ^5^HKS Architects, Dallas, TX, United States

**Keywords:** staging in place, aging in place, agile dwelling unit, information and communication technologies, older adult

## Abstract

As the number of older adults is growing rapidly in the U.S., the need for personalized, innovative, and sustainable Information and Communication Technologies (ICTs) solutions is critical to support individuals' social, emotional, and physical health. Such technology can significantly help older adults' ability to live independently in their homes despite the challenges the aging process may present, referred to as aging or staging in place. In this study, we explored ways to integrate ICTs into Agile Dwelling Units (AgDUs) through affordable, innovative, technology-enabled tools and practices that can be adapted to respond to individual's needs while supporting independent, secure, and engaged healthy living. The technology-enabled and human-centered AgDUs organically transform in response to users' needs. This approach offers a viable solution for older adults at different stages throughout their lifespan to transition into an intimate, technologically-enhanced living environment while allowing for (1) customization to user's needs; (2) cost optimization and maintenance; and (3) accessibility that minimizes gaps in compliance from a provider and user perspectives. Integrating ICTs in AgDUs to support health monitoring and management could reduce forthcoming pressure on the healthcare system and care providers to accommodate the needs of older adults. This approach is described through a collaborative multidisciplinary lens that highlights a partnership between academia, industry experts, and key stakeholders to advance healthy living and extend lifespan through design-build and technology integration. The main goal of this approach is to increase access to health services and optimize healthcare costs.

## Introduction

According to the World Health Organization (WHO), the proportion of the world's population over 60 years will nearly double from 12 to 22% from 2015 and 2050. By 2020, people aged 60 years and older have outnumbered children younger than 5 years. In addition, by 2050, 80% of older people will be living in low- and middle-income countries (1). This exponential growth in the older adult population is expected to create higher demand for care and health services and raise concerns about the adaptability of current environments to respond to this group's diverse needs. Undoubtedly, the demand for care is greater than the health services availability (2). Therefore, over the last few years, the older adult population's dependence on ICTs has risen to try and address this gap (3). Health technology is particularly critical for health monitoring and management for the older population. Health-centered digitalized technology lessens the burden on the growing aging population from how fast and frequently real-time data is updated, stored, and transmitted to a health portal where healthcare professionals can gather accurate patient progress (4). Some examples of health technologies can vary in functionality, such as wearable sensors, computerized cognitive tests, vital monitoring vitals biosensors, and automatic assistive bed lighting. The use of ICTs for supporting older adults' health is not exclusive to tasks directly related to disease treatment or medication management in clinical settings (5). ICTs, specifically Health ICTs, offer the possibility to help older adults in their residential settings with various everyday activities from digitalized technologies that store observed and analyzed data objectively from behaviors of movement all the way to progress of injuries with the use of internet, health applications, and remote monitoring devices, etc. (6). Such increased demand for ICTs to support older adults indicates the need for innovations and developments of ICTs that are designed to allow for independent living, along with a strategic and effective integration of technologies in residential units (7). In this study, we explored ways to integrate ICTs into Agile Dwelling Units (AgDU), a micro-dwelling unit specifically designed with older users in mind to support them in living an independent, secure, and engaged healthy life. AgDUs should at least be equipped with internet access and ideally connected to a health information system with user's primary healthcare. AgDUs may be any size, provided that the proposed total square footage is less than that of the primary unit and that local government requirements are satisfied (8, 9).

The study was conducted by a multidisciplinary team of experts, faculties, and students from the field of health technology, human factors, architecture, and urban planning. There were two phases: (1) research to identify the gaps and challenges in designing technology-integrated dwelling units for aging in place and (2) develop a prototype of agile dwelling units for aging in place. This article is focused on presenting the findings from phase one, which involved literature research, interviews, and roundtable discussions with experts. Eight interviews and two roundtable discussions were conducted remotely between October to December 2021. At least two researchers were involved in each session, one as a facilitator and the other as a note-taker. The meeting was conducted using Microsoft Teams and recorded for transcription and analysis. Participants were experts from research centers, non-profit organizations, design firms, universities, and corporations from various disciplines, such as computer science, public health, architecture, urban planning, nursing, health information technology, and digital technology. They were asked to share their expertise and provide perspectives in their relevant discipline. for better ICTs development that recognizes the need for older adult users' integration in the residential units to support successful (st)aging in place through an Agile Dwelling Unit (AgDU). For a sustainable long-term solution for the aging population as population growth is changing, AgDUs can help stabilize the healthcare system burdens, housing economy concerns, and lifestyle burdens as a result of aging.

## The state of ICT for aging in place

To age in place successfully, older adults must be able to live safely, independently, and comfortably in their area of residence while also participating in the community (10, 11). Researchers suggest that maintaining independence, including allowing aging adults to engage in various day-to-day activities, is important for healthy aging in place (12). As we age, we may need social and technical support to adapt to age-related changes and other factors that may interfere with our ability to perform daily activities independently. To understand the role of ICTs and the existing gaps and challenges, the research team conducted peer-reviewed literature research, discovery interviews, and roundtable discussions with experts from various fields, including medical professionals, academic scholars, and industry experts in Technology from Computer Science, Digital Health, Nursing, Health care, Smart Living, and Health Informatics.

Literature shows the increasing trend of ICT development as the economic growth from investments, price trends, and ICTs product revenues have overall positive outcomes (13, 14). However, there are still considerable concerns regarding the accessibility of ICTs for older adults, especially considering the complexity of technology functions and operations. A recent case study shows a lack of ICT skills while overreporting user competencies toward ICTs, which are inaccurate (15). A study conducted in Macedonia, Attica, Central Greece, and areas of Peloponnese had 300 participants aged 65–85 who were interviewed and surveyed. The study aimed to see how familiar elderly individuals were with modern technology. Overall, the study results showed that women, on average, had more exposure to smart appliances and health technology, such as refrigerators, electric stoves, and daily home appliances, while men used more TVs and ATMs more (16). Another qualitative research study in Hong Kong included 50 aging participants (ages 60 and older) surveyed on their negative outlooks on technology. Different technologies were categorized based on barriers experienced from a “personal” (i.e., related to health and functional capacities), “technological” (i.e., related to cost and complexity), and “environmental,” i.e., associated with the surrounding environment) perspective (17). Results suggested that individuals do not use technology due to lack of training, digital competency, economic factors, and that the devices are not user-friendly for this aging population. Lack of consideration for involving older adult users' characteristics and needs can impede the access, adoption, compliance, and continued use of ICTs.

Furthermore, interviews and roundtable discussions with industry experts in senior living and health technology highlighted the lack of ICT integration into residential units, especially for aging-in-place support. For instance, the Birkeland Current and Sovrinti company has developed and studied the movement of residents of senior living facilities using sensors and devices in Real Time Location System (RTLS). They collected total activity in a facility per day, time in or out of the home and associated durations, and room utilization (18). This technology has the potential as a preventative measure for community-dwelling older adults who may be in the early stages of dementia or are at risk of falling, but it has not been implemented yet. Using ICTs in residential units to support aging in place has much potential. Still, it needs to be designed to be reliable, easy to use, and adaptable to accommodate the changing needs of individuals.

## Lifelong approach to designing for older adults: Introducing (St)aging in place

Designing ICTs to support aging individuals must anticipate changes throughout the aging process. Such changes can be biological, physiological, environmental, psychological, behavioral, and social (19). These changes are noticeable when they impact older adults' health or ability to engage in various everyday activities. When age-related changes significantly affect older adults' independence to do basic daily activities, they need continued support at home, or they may need to move to other residential settings that provide care and support for them. However, the latter choice is less preferred, as most older adults want to stay in their current home and community for as long as possible (20).

When considering ways to accommodate older adults' changing needs, one should consider that aging in place is a dynamic adaptation process. Designing residential units or ICTs should focus on accommodating users with different needs and capacities. The development of the AgDU project presented in this article reconceptualizes the aging-in-place concept, which focuses on life during old age to the **(st)aging in place concept** that emphasizes the changing capacities of individuals throughout the life course as individuals experience aging (21). Integrating the stage concept instead of the age concept means implementing a multigenerational perspective focusing on a longer life- and health span (22). People in the same age group do not necessarily have the same physical, emotional, and cognitive characteristics and needs. Therefore, the staging perspective can capture different stages in life, making the design relevant for older adults and other age groups with various health statuses, capacities, and needs.

## Designing residential units with technology in mind

The (st)aging in place idea was further conceptualized into an Agile Dwelling Unit (AgDU) that is agile and adaptable to accommodate various health needs and users' challenges (23). This is a development of an existing residential typology called accessory dwelling units (ADU) that has grown in popularity as sources of secondary income, residences for aging relatives, and even primary residences for those seeking a minimalistic lifestyle. There are many benefits to building and residing in an accessory dwelling unit, such as increasing residential density, flexibility in site location, and the simplicity of spaces without loss of function. However, in the current accessory dwelling unit design and construction trend, these units are often rigid in terms of use and forms. This project explored the innovative solutions to the auxiliary dwelling unit and how they can be translated into an AgDU prototype by integrating ICTs, especially health technologies. In a nutshell, we define **AgDUs** as affordable, innovative, technology-enabled dwelling units that can be adapted to individual needs while supporting independent, secure, and engaged healthy living.

Regarding the spectrum of user interaction, the study performed by Demiris (24) organized ICTs into *active* and *passive* technology. When considering ICTs in relationship to residential units, the research team categorized ICTs based on the relation with the residential units: *attached* and *detached* technology.

- *Attached* technology comprises devices that are a part of a home infrastructure (usually built-in) and are not intended to travel outside the home. This includes lighting sensors, security cameras, digital home assistance, and smart home technology.
- *Detached* technology are devices that are not a part of the housing infrastructure and can become mobile such as mobile devices, tablets, and wearables.
- *Active* technology refers to devices that require the individual to operate and directly interact with the technology (24). For example, turning on and off the lights in a room *vs*. an automated light system where the individual does not have to operate a switch for the lights to function.
- *Passive* technology refers to devices that do not require an individual to operate directly (24). This technology is good for monitoring health and activity, which can be useful in determining users' declining physical or cognitive health. For example, using smart home sensors for detecting falls, tracking physical movements, and detecting heat for cooking. All these sensors do not require direct interaction, such as turning the sensor manually.

Table 1 illustrates the category and examples of ICTs that belong to the intersection of those categorizations. From the user's perspective of interacting with technology, these technologies are categorized as *active* and *passive*. *Active* technology needs direct interaction with the users, whereas *passive* technology is not operated directly by users. Concerning the physical environment, ICTs can be connected to parts of the building or be mobile or worn. Across the two categorizations, ICTs can be situated in four groups: *active-attached, active-detached, passive-attached*, and *passive-detached* technology. This matrix is intended to guide the identification and selection of ICTs that can be incorporated into residential units. The next step is to examine which technology is suitable for supporting older adults to (st)age in place regardless of their health status, physical limitation, health and technology/digital literacy, and desire.

**Table 1:** St(aging)-in-place ICT Matrix.

	**Attached** **(Part of the home infrastructure and not mare to travel outside the home)**	**Detached** **(Not built into the infrastructure of the home)**
**Active**	Ring doorbell Smart lights system	Pill dispenser Smartwatch
**Passive**	Fall detection sensor Real-time location sensor	Vital sign monitor

The matrix represents technology choices based on the relationship between the user, the built environment, and the behavioral needs.

## Discussion

Designing residential units with technology in mind offers viable solutions for older adults with various health conditions and physical capacities to maintain their independence when (st)aging in place. The categorization of ICTs for (st)aging in place allows designers, providers, and users to map the types of technology that can be integrated into the residential units. Identifying technology needs to be followed by strategies to support successful technology adoption and continued use by older adults and other residents (if any) in the residential units. Literature research, interviews, and roundtable discussions with experts have demonstrated the need for non-invasive, ubiquitous, non-interactive, and simplified functional ICTs to support the everyday life of older adults, including health management and monitoring. This research aims to identify the most effective health ICTs for the aging population. Informed by the findings, the research team proposes three strategies for a successful transition to technology-integrated residential units for (st) aging in place:

### Strategy 1: Customizability of ICTs to users' needs

A life-long approach should prepare for the ever-changing health and capacities of users. The physical environment and integrated technology should be customized to the user's needs, abilities, and desires. For instance, customizable displays of household appliances (i.e., font and icon size, notification volume, and methods to input commands) allow older adults with different capacities to operate those appliances for housekeeping activities. Moreover, it is important to remember that customizing technology should involve needs assessment based on users' demographics, digital, and health literacy. This type of customizability is aligned to the findings suggested by Ferati et al. (25), where the sustainability of technology long term uses “Systems Thinking” and “Designing Thinking” to identify several challenges in design solutions (25). Systems thinking allows more for holistic methodology and encompasses the understanding of inter-relationships such as aging and the use of technology. Design thinking encompasses challenging the current status over human ergonomics and technology integration. A ‘personalized aging' approach allows users to modify the infrastructure to match their needs and desires across the life stages.

### Strategy 2: Cost optimization and maintenance

ICTs are costly, especially when designing AgDU with multiple forms of technology. What makes ICTs expensive involves three aspects: the initial purchase, maintenance of the ICTs through continuous financial investments or services that come with ICT products, and the need to change the technology. Finding strategies to best implement training goes hand in hand with what services are being provided and the type of ICT used. For example, remote monitoring devices linked to an app, such as a biomarker that sends vital signs or trackable steps to the application, have been used after a patient has been discharged. In the long term, this can reduce patients being readmitted to the hospital and reduce costs that could be avoided with more real-time data tracking. This would increase more accurate, productive, optimal medical consultations and decrease unnecessary doctor and medical referrals to see other specialists. Due to COVID-19, tracking certain factors of an individual's health using real-time data is developing into the main health-centric feature changing how health services and medical consultations are done (26). Similarly, a mHealth intervention of 1,617 individuals with diabetes mellitus. The study proposes cost optimization through diabetes and insulin management through smartphone devices. The systems would include clinician consultations frequently, usage, messaging, mobile applications, and addressed low-income patients (27). Results showed cost reductions from avoided unnecessary visits to the hospital, self-monitoring, are cost-effective, and promote lifestyle changes that reduce costs long term, such as healthy dieting (27).

### Strategy 3: Accessibility to minimize gaps in compliance

During the roundtable, digital technology experts emphasized ontraining and educating the user about how to use the technology and its functionalities. Users will have higher levels of compliance when they are exposed to training, personalized sessions (online or in-person), and reward systems added for apps or technologies that needs sustainable self-reporting from the users (28). Additionally, when placing different ICTs in a residential unit, it is important to consider the visibility, proximity to related activity, and the ease of access so that users are indirectly reminded to continue using them appropriately.

The three strategies align with the theoretical model of Maslow's Hierarchy of needs. The use of a device can become a habit, and the use of the device can be out of preference or necessity. Human factors and ergonomics are used to identify the health necessities that technology can provide. Maslow's model is a model that determines the order in which the needs of a human are being met. There are five stages of needs: physiological needs, safety, love and belonging, esteem, and self-actualization (29). The first stage is the physiological needs of a human; food, water, and shelter. This project aims to provide all these needs, but most importantly, health under the category's Physiological needs, Safety and Security using technology (29). For physiological need, such as physical mobility, cognitive support, and maintaining or reducing symptoms of chronic diseases of an individual, several attached technology systems cover multiple needs for aging in place.

**Figure 1:**
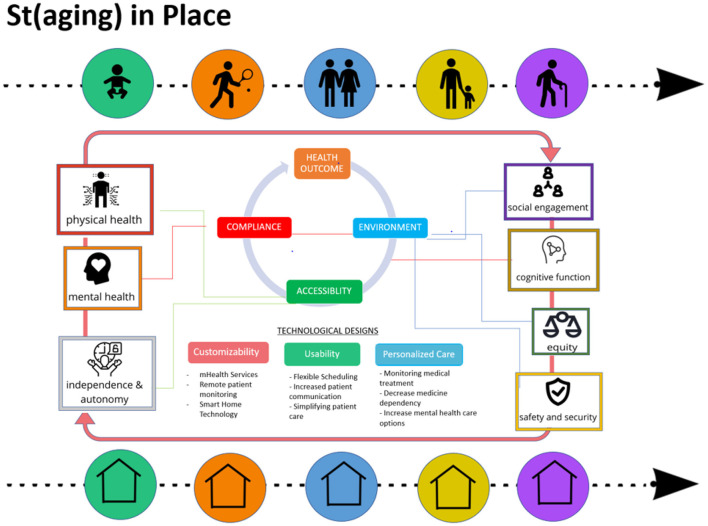
St(aging)-in-place ICT strategy diagram. The (st)aging in place concept highlights the home environment's capability to respond to the needs of the individual throughout different life stages in a single residence. The diagram depicts how technological designs support environment, accessibility, and compliance factors.

AgDUs provide physiological needs through the support of health ICTs for all physical or cognitive health-related behaviors highlighted in Figure 1. For example, Cognitive computerized testing can be used as a tactical format to manage dementia, wearable sensors for evaluating walking abilities or sleeping disorders, and environmental sensors to assess the risk of falls (30).

Another instance of physiological need that AgDU can provide is sustainable and affordable shelter, especially when an older adult may develop a chronic disease that does not allow for work in their later stages of life. An experience such as a fall can potentially go from walking to relying on a wheelchair. The AgDU's modality would comply with wheelchair accessibility needs by designing a first floor, wide, smooth flooring. Safety and security needs are also considered from having a sustainable residential environment being affordable of Maslow's model. These three strategies are highlighted in Figure 1. The three strategies are categorized as compliance, accessibility, and environment. Within each category are design factors, customizability, usability, and personalized care, with a few examples of how the design factors support the three strategies mentioned above. The three strategies and the three design factors are designated to one another in a color-coordinated fashion. The pictures show aspects of health such as physical health, mental health, cognitive function, etc., which need to be considered when integrating various ICTs to achieve better health outcomes while minimizing the burdens on the healthcare system — i.e., compliance, accessibility, and the environment.

As mentioned in the “St(aging)-in-Place” section, the concept comes from the reconceptualization of “aging in place” with the notion of continuous accommodations in the residential space of an aging individual's needs. As the residential environment changes, health ICTs are supposed to be a support and part of the accommodations as needed among older adults. This concept is highlighted in Figure 1 (i.e., both arrows with circles on top and the bottom of the diagram shows a period over time of “stages” in life changing with a same residential environment to indicate that an aging individual does not need to move away from their home to satisfy whatever needs are required to sustain independent living).

## Conclusion

In recent years innovation in Information Technology (ICT) has increased overall. Even so, there is a need to improve ICT design for older adults and the services provided for using ICTs to enhance digital competency and digital literacy among older adult users, specifically for health technological design. Residential units designed with the integration of ICTs in mind offer a viable solution for older adults to stage in place. With the variety of health conditions, physical capacities, and availability of social support, a technology-integrated residential unit like AgDU can facilitate users in adapting to the changes throughout their life course. Collaboration between users, architects, healthcare, and technology providers is needed to ensure successful integration into the residential units and care plans while providing reliable support for the users for successful technology adoption and continued use to support their health and wellbeing. This study is an early ideation stage of an AgDU. While several early prototypes have been designed and developed by the University of Texas at Arlington students, there is a need to expand the coalition project to study and test the AgDU concept in the community. Involving government agencies and industry partners is a critical next step to further developing AgDU design strategies, development, and deployment.

Additionally, further research is needed to explore the integration of ICTs beyond the physical and digital spaces. This would allow for coordinated care efforts among older adults, caregivers, healthcare providers, and other supporting services contributing to the staging in place. Overall, studies and metrics offer significant promise for the future of aging through ICT skills improvements and design in the built environment.

## Data availability statement

The original contributions presented in the study are included in the article/supplementary material, further inquiries can be directed to the corresponding author.

## Author contributions

All authors listed have made a substantial, direct, and intellectual contribution to the work and approved it for publication.
